# A three amino acid deletion in the transmembrane domain of the nicotinic acetylcholine receptor α6 subunit confers high-level resistance to spinosad in *Plutella xylostella*

**DOI:** 10.1016/j.ibmb.2016.02.001

**Published:** 2016-04

**Authors:** Jing Wang, Xingliang Wang, Stuart J. Lansdell, Jianheng Zhang, Neil S. Millar, Yidong Wu

**Affiliations:** a College of Plant Protection, Nanjing Agricultural University, Nanjing, China; b Department of Neuroscience, Physiology & Pharmacology, University College London, London, United Kingdom

**Keywords:** *Plutella xylostella*, Spinosad, Nicotinic acetylcholine receptor, Deletion, Insecticide resistance

## Abstract

Spinosad is a macrocyclic lactone insecticide that acts primarily at the nicotinic acetylcholine receptors (nAChRs) of target insects. Here we describe evidence that high levels of resistance to spinosad in the diamondback moth (*Plutella xylostella*) are associated with a three amino acid (3-aa) deletion in the fourth transmembrane domain (TM4) of the nAChR α6 subunit (Pxα6). Following laboratory selection with spinosad, the SZ-SpinR strain of *P. xylostella* exhibited 940-fold resistance to spinosad. In addition, the selected insect population had 1060-fold cross-resistance to spinetoram but, in contrast, no cross-resistance to abamectin was observed. Genetic analysis indicates that spinosad resistance in SZ-SpinR is inherited as a recessive and autosomal trait, and that the 3-aa deletion (IIA) in TM4 of Pxα6 is tightly linked to spinosad resistance. Because of well-established difficulties in functional expression of cloned insect nAChRs, the analogous resistance-associated deletion mutation was introduced into a prototype nAChR (the cloned human α7 subunit). Two-electrode voltage-clamp recording with wild-type and mutated nAChRs expressed in *Xenopus laevis* oocytes indicated that the mutation causes a complete loss of agonist activation. In addition, radioligand binding studies indicated that the 3-aa deletion resulted in significantly lower-affinity binding of the extracellular neurotransmitter-binding site. These findings are consistent with the 3-amino acid (IIA) deletion within the transmembrane domain of Pxα6 being responsible for target-site resistance to spinosad in the SZ-SpinR strain of *P. xylostella*.

## Highlights

• Spinosad resistance in SZ-SpinR is recessive and autosomal.
• A 3-aa deletion in TM4 of Pxα6 tightly linked to spinosad resistance.
• The deletion mutation causes a complete loss of function of in a prototype nAChR (Hα7).

## Graphical abstract

**Figure:**
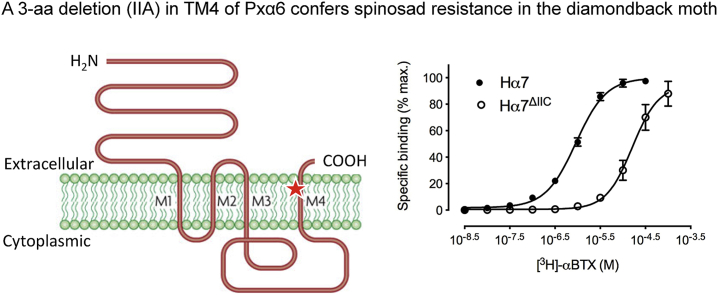

## 1 Introduction

The spinosyns are a family of secondary metabolites from the aerobic fermentation of the soil bacterium *Saccharopolyspora spinosa* on nutrient media. They show potent insecticidal activities against a broad spectrum of pest insects (Thompson et al., 2000). Spinosad, the first commercial product of spinosyns, is a mixture of spinosyn A (the major component) and spinosyn D (the minor component). Spinetoram is a semi-synthetic spinosyn, which is developed by making two synthetic modifications to fermentation-derived spinosyns (Kirst, 2010, Dripps et al., 2011). Compared with spinosad, spinetoram has improved insecticidal efficacy and a broader pest insect spectrum (Geng et al., 2013). The mode of action of spinosyns involves a binding site on the nicotinic acetylcholine receptors (nAChRs) that is distinct from that targeted by neonicotinoids (Millar and Denholm, 2007, Watson et al., 2010, Sparks et al., 2012, Geng et al., 2013).

Genetic and chemical mutagenesis studies with the fruit fly, *Drosophila melanogaster* showed that there is a causal link between loss of function mutations of the *Drosophila* nAChR *Dα6* gene and high levels of resistance to spinosad (Perry et al., 2007, Watson et al., 2010, Perry et al., 2015), demonstrating that the Dα6 subunit of *D. melanogaster* is the primary target site of spinosad. The association of spinosad resistance with loss of function mutations of a homolog of Dα6 has been reported in the diamondback moth, *Plutella xylostella* (Baxter et al., 2010) and the oriental fruit fly, *Bactrocera dorsalis* (Hsu et al., 2012). Recently, point mutations in nAChR α6 subunits were reported to confer resistance to spinosad in several insect species. A point mutation (G275E) located at the top of the third transmembrane domain (TM3) of the nAChR α6 subunit in the western flower thrip, *Frankliniella occidentalis* has been suggested to cause resistance through abolishing the modulatory effects of spinosad (Puinean et al., 2013). Interestingly, the G275E mutation has also been detected in the spinosad-resistant melon thrip, *Thrips palmi* (Bao et al., 2014). In addition, a point mutation (P146S) close to the conserved Cys-loop in Dα6 contributes to moderate-level and incompletely dominant resistance to spinosad in *D. melanogaster* (Somers et al., 2015).

The diamondback moth, *P. xylostella* is a global pest insect of cruciferous vegetables. This pest has evolved resistance to almost all classes of insecticides including newer chemistries, such as indoxacarb, abamectin, emamectin benzoate, spinosad and chlorantraniliprole (Zhao et al., 2002, Zhao et al., 2006, Pu et al., 2010, Wang and Wu, 2012 Wang et al., 2016a). After only a few years of extensive use, resistance to spinosad in *P. xylostella* emerged in Hawaii (2000), Georgia (2001), and California (2001) of the United States and has resulted in field control failures (Zhao et al., 2002, Zhao et al., 2006). Recently, low levels of resistance to spinosad (10- to 35-fold) have been detected in some field populations of *P. xylostella* from Pakistan (Khaliq et al., 2007) and China (Pu et al., 2010, Xia et al., 2014, Jiang et al., 2015).

The spinosad resistance (13,100-fold) in the Pear-Sel strain of *P. xylostella*, selected from a field-collected population in Hawaii of the United States, was inherited as a single, autosomal recessive trait (Zhao et al., 2002). A genetic mapping approach has recently revealed that Pxα6, the nAChR Dα6 homologue, was genetically linked to the spinosad resistance derived from Pear-Sel (Baxter et al., 2010). A mutation in the 5′ donor site of intron 9 causes mis-splicing of Pxα6 mRNA and thereby introduces a premature stop codon between TM3 and TM4 (Baxter et al., 2010, Rinkevich et al., 2010). In the present study, a Chinese strain of *P. xylostella* (SZ-SpinR) developed ∼ 1000-fold resistance to spinosad as a result of laboratory selection. A 3-amino acid deletion in TM4 of Pxα6 was fixed in the SZ-SpinR strain and was tightly linked with recessive resistance to spinosad. The analogous deletion mutation was introduced into human nAChR α7 subunit, and heterologous expression studies demonstrated that the 3-amino acid deletion in TM4 caused a complete loss of receptor function and altered the affinity of ligand (α-bungarotoxin) binding. Our findings support the conclusion that insect nAChRs containing the α6 subunit are the major target site for spinosad, and suggest that insects may have diverse ways to produce loss of function mutations and thereby confer resistance to spinosad.

## 2 Materials and methods

### 2.1 Insect strains

The susceptible strain SZ was originally collected from Shenzhen, Guangdong Province, China, in May 2002. SZ has been reared in laboratory since then without exposure to any insecticides. The SZ-SpinR strain was derived from SZ with continuous selection with spinosad for more than 80 generations until resistance was stabilized.

All stages of the two strains were reared on radish seedlings (*Raphanus sativus* L.) at 25 ± 1 °C, 65 ± 5% relative humidity (RH), and a photoperiod of 16: 8 (L:D) h. Adults were fed with 10% honey solution and allowed to lay eggs on radish seedlings.

### 2.2 Insecticides and chemicals

Three formulated insecticides used for bioassays included spinosad (25 g L^−1^ SC, Dow AgroSciences, Shanghai, China), spinetoram (60 g L^−1^ SC, Dow AgroSciences, Shanghai, China), and abamectin (20 g L^−1^ EC, Guangdong Academy of Agricultural Sciences, Guangzhou, China). The non-ionic detergent Triton X-100 was purchased from Beijing Solarbio Science and Technology Co. Ltd. (Beijing, China).

### 2.3 Leaf dip bioassay

Formulated insecticides were diluted to generate five to seven serial dilutions with distilled water containing 1 g L^−1^ Triton X-100 (to facilitate uniform treatment of the plant tissues with the active ingredient). Cabbage (*Brassica oleracea*) leaf discs (*diameter* = 6.5 cm) were cut and dipped in an insecticide solution for 30 s. Control discs were treated with 1 g L^−1^ Triton X-100 solution only. The leaf discs were dried at room temperature for about 2 h. One treated leaf disc with ten third instar larvae was placed in a plastic petri dish, then kept at 25 ± 1 °C and an RH of 65 ± 5% with a photoperiod of 16:8 (L:D) h. For each concentration, 40 third instar larvae were treated. Mortality was assessed after 48 h. Larvae were considered dead if they could not move when probed with a camel hair brush. Bioassay data were analysed using Poloplus^®^ software (LeOra Software, Berkeley, CA).

### 2.4 Genetic crosses

Female and male fourth-instar larvae of the spinosad-resistant SZ-SpinR and susceptible SZ strains were visually separated according to Liu and Tabashnik (1997). Virgin female moths from the SZ-SpinR strain were mass-crossed with male moths of the SZ strain, and vice versa. F_1_ progeny from the reciprocal crosses were pooled and mass-crossed to produce F_2_ progeny. Sixty third instar larvae of the F_2_ progeny were treated with 10 mg L^−1^ of spinosad. After 48 h treatment, both dead and survivors were collected and frozen in −20 °C for *Pxα6* genotyping.

At least 100 adults of each sex were used in all mass crosses. The toxicological responses to spinosad of F_1_ offspring from the reciprocal crosses and the F_2_ progeny were determined using the leaf dip bioassay described earlier.

### 2.5 Cloning of full-length cDNAs of the nAChR α6 subunit of *P. xylostella* (Pxα6)

Total RNA of 10 fourth-instar larvae was extracted for each strain using the SV Total RNA Isolation System Kit (Promega, Madison, WI, USA) according to the manufacturer's instructions. First-strand cDNA was synthesized from 2 μg total RNA using an oligo(dT)_15_ primer and M-MLV reverse transcriptase (Promega). A pair of specific primers (forward: 5′- AGCCATGGCCGTGCTGCTAGCGG-3′, reverse: 5′-CGGCTCGGTGCAGTCCGCTCACT-3′), designed based on two full-length cDNAs of *Pxα6* deposited in GenBank (GU207835 and GQ247883), were used to amplify the complete coding cDNAs of *Pxα6* from the SZ and SZ-SpinR strains by polymerase chain reaction (PCR). The PCR solution consisted of 12.5 μL 2 × PCR reaction buffer, 1 μL of each primer (10 μM), 0.5 μL Tks Gflex™ DNA Polymerase (TAKARA, Dalian, China), 1 μL cDNA template and 9 μL sterile distilled water in a final volume of 25 μL. The amplification was performed at 95 °C for 1 min and 35 cycles of 98 °C for 10 s and 68 °C for 1 min. The PCR products of the expected size (about 1500 bp) were purified from low melting point agarose gel using a Wizard PCR Preps DNA kit (Promega) and then ligated into a pGEM-T easy vector (Promega). The target cDNA fragments were sequenced by Life Technology (Shanghai, China). To determine the frequency of *Pxα6* transcript isoforms, 25 clones from the SZ sample and 34 clones from the SZ-SpinR sample were sequenced.

### 2.6 DNA-based genotyping for Pxα6

Genomic DNAs of individual dead or survival larvae were extracted using the AxyPrep™ Multisource Genomic DNA Miniprep Kit (Axygen Biosciences, Union, CA) according to the manufacturer's protocol and used as the template for PCR. The forward primer (5′-TTGATGACAGTGATTGTGTGTGTT-3′) and the dye-labelled (FAM) reverse primer (5′-TCACTGCACGATGATGTGCGG-3′) were used to amplify a 112 bp fragment flanking the 9-bp deletion site in TM4 of Pxα6. The PCR products tagged with dye were subsequently separated by electrophoresis on the ABI 3130 capillary sequencer. The genotypes of *Pxα6* were determined according to the resulted sequence length diagrams.

### 2.7 Site-directed mutagenesis

Site-directed mutagenesis was performed on the human nAChR α7 subunit cDNA (Hα7) in plasmid expression vector pcDNA3 (pcDNA3-Hα7) using the QuikChange mutagenesis kit (Stratagene, La Jolla, CA) and was verified by nucleotide sequencing. Nine nucleotides encoding amino acids IIC (analogous to the IIA deletion in Pxα6) were deleted to create plasmid pcDNA3-Hα7^ΔIIC^.

### 2.8 Two-electrode voltage clamp recording

*Xenopus laevis* oocytes were isolated and defolliculated, as described previously (Young et al., 2008). Expression of the recombinant human α7 nAChR was achieved by manual injection into the oocyte nucleus of plasmid expression vector (10–30 ng) encoding the wild-type or mutated human nAChR α7 subunit cDNA (pcDNA3-Hα7 and pcDNA3-Hα7^ΔIIC^, respectively). Oocytes were injected with a volume of 32.2 nl using a Drummond variable volume micro-injector. Two electrode voltage-clamp recordings were performed as described previously (Young et al., 2008). Agonists were applied to voltage-clamped oocytes using a computer-controlled gravity perfusion system (ALA Scientific Instruments, Farmingdale, NY).

### 2.9 Cell culture and transfection

The mammalian cell line tsA201, derived from the human embryonic kidney 293 cell line, was obtained from Dr. William Green (University of Chicago, Chicago, IL). Cells were cultured in Dulbecco's modified Eagle's medium (Invitrogen, Paisley, UK) containing 2 mM L-GlutaMAX (Invitrogen) plus 10% heat-inactivated fetal calf serum (Sigma, Poole, UK) with penicillin (100 U/ml) and streptomycin (100 μg/ml) and were maintained in a humidified incubator containing 5% CO_2_ at 37 °C. Cells were transiently transfected using Effectene transfection reagent (Qiagen, Crawley, UK) according to the manufacturer's instructions. Cells were transfected with plasmid expression vector encoding the wild-type or mutated human nAChR α7 subunit cDNA (pcDNA3-Hα7 and pcDNA3-Hα7^ΔIIC^, respectively). As has been shown previously, efficient folding and expression of α7 nAChR in non-neuronal cell lines such as tsA210 requires co-expression with the chaperone protein RIC-3 (Lansdell et al., 2005, Millar, 2008). For this reason, plasmids encoding Hα7 and Hα7^ΔIIC^ were co-transfected a plasmid (pcDNA3-CeRIC3) containing the *C. elegans* RIC-3 cDNA (Halevi et al., 2002). Cells were transfected overnight and assayed for expression approximately 40–48 h after transfection.

### 2.10 Radioligand binding

[^3^H]-α-bungarotoxin ([^3^H]-αBTX; 56 Ci/mmol; Tocris Bioscience, Bristol, UK) was a gift from Syngenta (Bracknell, UK). Radioligand binding to transiently transfected tsA201 cells was performed essentially as described previously (Lansdell and Millar, 2004). Transfected cells were resuspended in Hank's buffered saline solution (Gibco, Paisley, UK) containing 1% BSA and incubated with [^3^H]-αBTX for 2 h at room temperature in a total volume of 300 μl. Non-specific binding was determined in the presence of nicotine (1 mM) and carbamylcholine (1 mM). Saturation binding experiments were performed by incubating triplicate samples of transfected cells with varying concentrations of [^3^H]-αBTX. Radioligand binding was assayed by filtration onto Whatman GF/A filters (pre-soaked in 0.5% polyethylenimine), followed by rapid washing with phosphate-buffered saline (Oxoid, Basingstoke, UK) using a Brandel cell harvester. Bound radioligand was quantified by scintillation counting. Curves for equilibrium binding were fitted using GraphPad Prism (GraphPad Software, San Diego, USA).

## 3 Results

### 3.1 Characterization of cross-resistance and inheritance mode

The spinosad-selected SZ-SpinR strain exhibited 940-fold resistance to spinosad compared with the susceptible SZ strain. The SZ-SpinR strain developed 1060-fold cross-resistance to spinetoram (a semi-synthetic spinosyn), but no cross-resistance to abamectin (Table 1). LC_50_s of spinosad against the F_1_ progeny from reciprocal crosses between SZ-SpinR and SZ were very similar (0.84 and 0.88 mg L^−1^) and close to that of the susceptible SZ strain (0.27 mg L^−1^), suggesting that the spinosad resistance in the SZ-SpinR strain was inherited as an autosomal and incompletely recessive trait (Table 1).

### 3.2 Identification of a 3-aa deletion in TM4 of Pxα6 in the SZ-SpinR strain

**Fig. 1:**
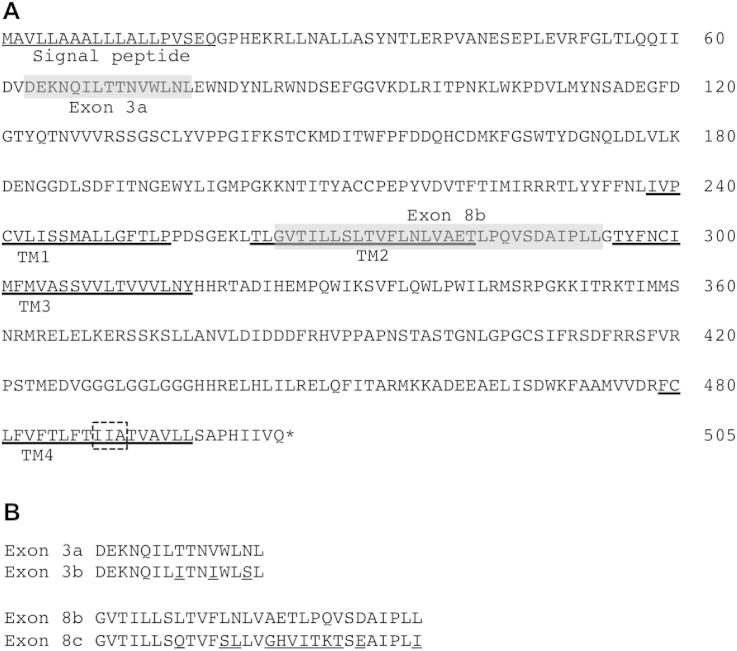
Amino acid sequence of *Plutella xylostella* nicotinic acetylcholine receptor (nAChR) Pxα6 (GenBank nos. KU130399 for SZ, and KU130400 for SZ-SpinR). A. The predicted four transmembrane domains are underlined in bold (TM1-TM4). Two alternative splice exons 3a and 8b are marked in grey. The deleted three amino acids in the resistant SZ-SpinR strain are boxed with dashed line. B. Amino acid sequence alignment of two alternative splice exons 3a/3b and 8b/8c. The different amino acids were underlined between two variants of exon 3 or exon 8.

**Fig. 2:**
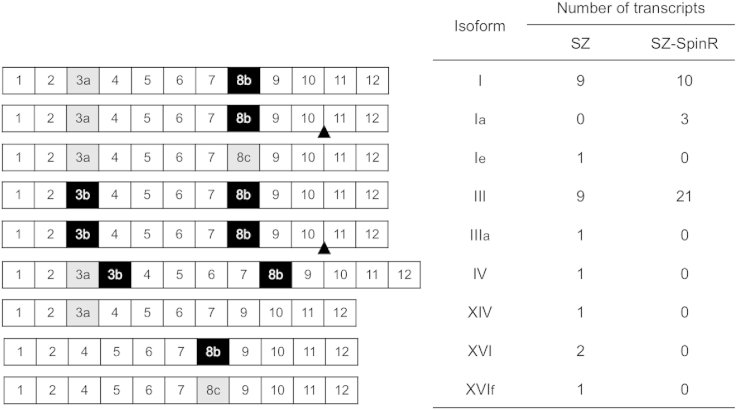
Schematic diagrams and frequency of variable isoforms of *Plutella xylostella* nicotinic acetylcholine receptor (nAChR) Pxα6 from the susceptible SZ and spinosad-resistant SZ-SpinR strains. Exons are represented by the numbered boxes. The black triangle indicates the location of a 30 bp insertion (CCTAACTAACGTGAGTGTCATCGGGCCCAG) between Exon 10 and Exon 11. Isoforms were numbered according to prior conventions (Grauso et al., 2002, Rinkevich and Scott, 2009). Frequency of each isoform was listed to the right of each diagram for the SZ (n = 25) and SZ-SpinR (n = 34) strains.

The complete coding cDNA sequences of *Pxα6* from the resistant SZ-SpinR and susceptible SZ strains were successfully amplified, cloned and sequenced. As previous studies showed (Baxter et al., 2010, Rinkevich et al., 2010), alternative exons were observed for exon 3 and exon 8 (Fig. 1), and considerable splice-form variation was detected in both strains (Fig. 2). All spliced transcripts of *Pxα6* detected from both strains have complete open reading frames. Isoforms I and III (containing exons 3a/8b and 3b/8b, respectively; see Fig. 2) are the predominant transcripts in both strains.

In contrast to all complete coding cDNA variants (n = 25) obtained from the susceptible strain, all *Pxα6* variants (n = 34) sequenced from the spinosad-resistant SZ-SpinR strain had a 9-bp in-frame deletion in exon 12, resulting in loss of 3 amino acids (IIA) in TM4 (Fig. 1). The three deleted amino acids in TM4 of Pxα6 are highly conserved among different species of insect, indicating there may be a role for this deletion in determining the nAChR function and thereby spinosad resistance.

### 3.3 Linkage between the 9-bp deletion and spinosad resistance

**Fig. 3:**
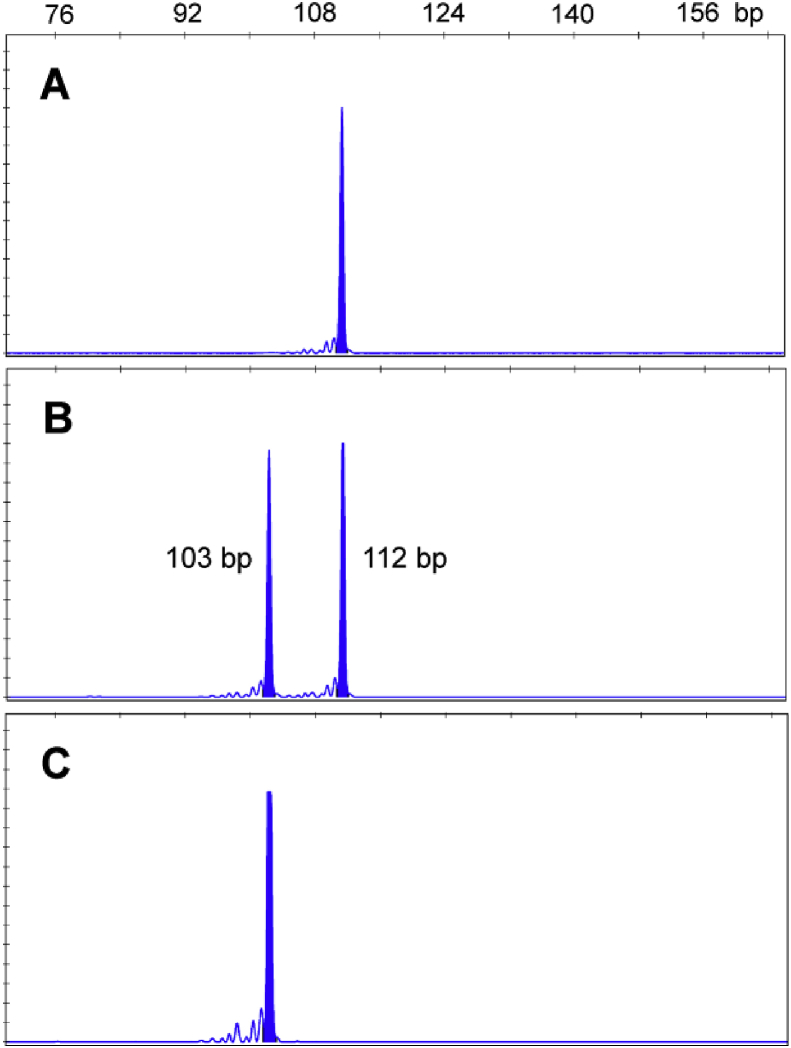
Sequence length diagram of the fragment flanking the 9-bp deletion site in the TM4 of Pxα6. (A) Homozygote for the wild genotype. (B) Heterozygote. (C) Homozygote for the 9-bp deletion genotype.

Comparison of cDNA and genomic DNA sequences, indicated that a 9-bp deletion within exon 12 of the *Pxα6* gene was responsible for the deletion identified in the cloned cDNAs. Thus, a DNA-based genotyping method was developed (Fig. 3).

To investigate the association of the 3-aa deletion in TM4 of Pxα6 with spinosad resistance, a set of genetic crosses was performed as described in the methods. Sixty third instar larvae from the F_2_ progeny were treated with a diagnostic concentration of spinosad (10 mg L^−1^) and after 48 h of treatment both survivors and dead larvae were genotyped. Genotyping results (Table 2) revealed that all 13 survivors were homozygous for the 3-aa deletion of Pxα6, and that the 47 dead larvae were either heterozygous (32 of 47) or homozygous for the wild type (15 of 47). This result demonstrates that there is highly significant, tight genetic linkage between the 3-aa deletion mutation and spinosad resistance in the SZ-SpinR strain (P < 0.0001, Fisher's exact test), and confirms that the 3-aa deletion mutation is recessive.

### 3.4 Influence of the 3-aa deletion on nAChR function

**Fig. 4:**
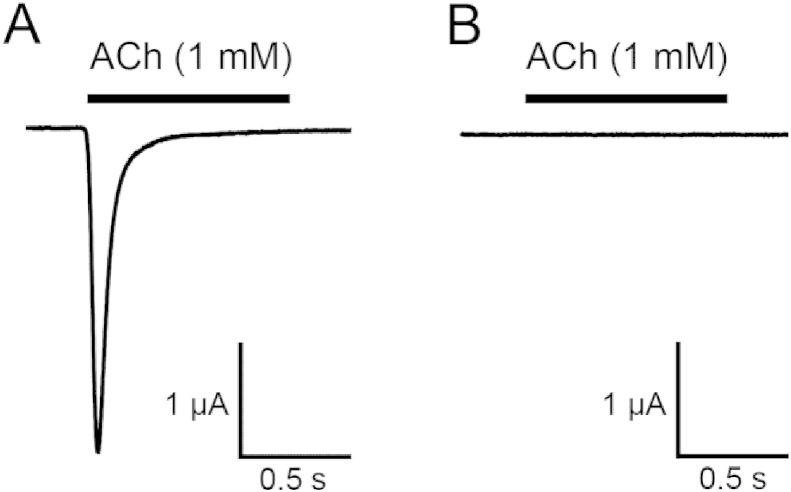
Human α7 nAChRs containing a three amino acid deletion (Hα7^ΔIIC^), analogous to the IIA deletion in Pxα6, are non-functional. Representative traces are shown illustrating functional responses to acetylcholine in wild-type α7 (A) and the absence of response with mutated Hα7^ΔIIC^ (B). The lack of functional expression was observed in studies with more than 50 independent oocytes.

Considerable difficulties have been encountered in the functional expression of recombinant insect nAChRs in heterologous expression systems (Millar, 2009, Millar and Lansdell, 2010). For this reason, the analogous three amino acid deletion was introduced by site-directed mutagenesis into a more easily expressed prototype nAChR subunit, the human nAChR α7 subunit. This is a strategy that has previously been used successfully to examine the functional properties of a spinosad-associated point mutation (G275E) in the α6 nAChR cloned from *F. occidentalis* (Puinean et al., 2013). Three amino acids in the α7 TM4 domain (IIC), at positions analogous to amino acids IIA in Pxα6, were deleted by site-directed mutagenesis. Wild-type and mutated α7 nAChR was expressed in *Xenopus* oocytes using well-established methods and examined by two-electrode voltage clamp recording. As has been reported previously (Peng et al., 1994), wild-type human α7 nAChRs produced rapidly desensitizing inward currents in response to acetylcholine. In contrast, no agonist-evoked currents were detected with the mutated Hα7^ΔIIC^ subunit (Fig. 4). This finding suggests that deletion of three amino acids (IIC) from the TM4 domain of Hα7 results in a non-functional nAChR.

**Fig. 5:**
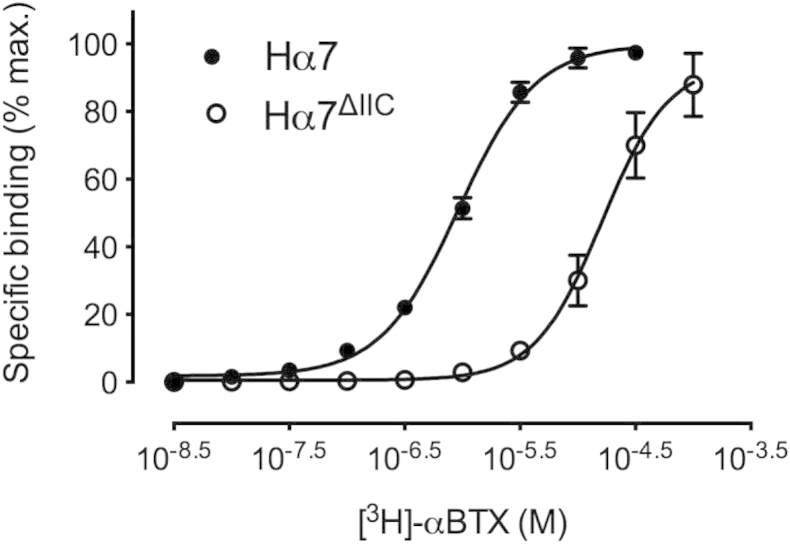
Binding of [^3^H]-αBTX to tsA201 cells transfected with human α7 nAChRs. Similar levels of specific cell-surface radioligand binding were observed in cells transfected with wild-type (Hα7) and mutated (Hα7^ΔIIC^) human α7 nAChR subunit. However, [^3^H]-αBTX bound with a significantly lower affinity (P = 0.03) to the Hα7^ΔIIC^ (EC_50_ = 0.9 ± 0.1 nM) than to Hα7 (EC_50_ = 20.0 ± 5.8 nM). Data points are means (±SEM) of three independent experiments, each with triplicate samples.

Despite the absence of functional expression, radioligand binding was employed to examine whether nAChRs could be detected in transiently transfected cultured cells expressing wild-type and mutated Hα7. Specific cell-surface binding of [^3^H]-αBTX was detected in cells transfected with both wild-type (Hα7) and mutant (Hα7^ΔIIC^) subunit cDNAs (Fig. 5). Similar levels of total binding were observed for wild-type and mutated Hα7 using saturating concentrations of [^3^H]-αBTX. However, despite similar levels of total cell-surface binding, [^3^H]-αBTX bound with a significantly lower affinity (P = 0.03) to the mutated receptor (EC_50_ = 0.9 ± 0.1 nM) than to the wild-type receptor (EC_50_ = 20.0 ± 5.8 nM) (Fig. 5).

## 4 Discussion

We have identified high levels of resistance to spinosad in a laboratory-selected strain of the diamondback moth, *P. xylostella*. By molecular cloning, we have demonstrated that resistance is associated with a 3-aa deletion in the fourth transmembrane domain of the nAChR α6 subunit (Pxα6). These findings extend previous studies that have reported target-site resistance to be associated with mutations in nAChR α6 subunit of other insect species (discussed in more detail below). Similarly, target-site resistance to neonicotinoid insecticides has been reported to be associated with target-site mutations in other insect nAChR subunits (Liu et al., 2005, Bass et al., 2011).

Because of the well-characterised difficulties associated with the functional expression of cloned insect nAChRs (Millar, 2009, Millar and Lansdell, 2010), we adopted an approach that has been used previously (Puinean et al., 2013) to introduce an analogous mutation in a prototype nAChR, the human α7 nAChR. Our findings, indicating that introduction of the 3-aa (IIC) deletion into Hα7 results in a loss of function suggest that the resistance-associated IIA deletion in Pxα6 may also result in a non-functional nAChR. This would be consistent with previous studies that have reported that deletion of the gene encoding the nAChR α6 subunit in *Drosophila* is associated with resistance to spinosad (Perry et al., 2007) and that over-expression of insect α6 subunit cDNAs in *Drosophila* can confer sensitivity to spinosad (Perry et al., 2015).

In addition to electrophysiological studies, radioligand binding was performed with [^3^H]-αBTX, a nAChR antagonist that binds competitively with acetylcholine at its extracellular binding site. The fact that similar levels of cell-surface radioligand binding were observed in cells transfected with wild-type (Hα7) and mutated (Hα7^ΔIIC^) nAChRs suggests that the lack of functional expression is not a consequence of the 3-aa deletion resulting in an inability to express nAChR subunit protein. However, the finding that [^3^H]-αBTX binds with significantly lower affinity to mutated (Hα7^ΔIIC^) nAChRs indicates that the presence of the 3-aa transmembrane deletion can exert long-range effects at the extracellular agonist binding site.

Spinosad has chemical similarity to ivermectin, another widely-used macrocyclic lactone pesticide (Wolstenholme and Kaplan, 2012). Previous X-ray crystallisation studies have identified a transmembrane binding site for ivermectin in the glutamate-gated chloride channel (GluCl) (Hibbs and Gouaux, 2011), a pentameric receptor with close structural similarity to nAChRs. Although the precise binding site for spinosad in nAChRs is not known, it seems plausible that spinosad may bind at a location in the transmembrane domain of nAChR that is similar to the known binding site for ivermectin in GluCl. For example, studies with artificial subunit chimeras are consistent with spinosad interacting with the transmembrane domain of the nAChR α6 subunit (Somers et al., 2015). Further support is provided by evidence that a spinosad resistance-associated point mutation (G275E) identified in the nAChR α6 subunit of *F. occidentalis* (Puinean et al., 2013) is located at a position that is analogous to an amino acid in GluCl that forms a direct van der Waals interaction with ivermectin (Hibbs and Gouaux, 2011). However, in contrast, the three amino acids in GluCl that are analogous to the IIA deletion in TM4 of Pxα6 do not form direct interactions with ivermectin (Hibbs and Gouaux, 2011). Instead, ivermectin makes direct contact (by hydrogen bonding and van der Waals interactions) with TM1, TM2 and TM3 (Hibbs and Gouaux, 2011). This is consistent with evidence from our radioligand binding studies that the 3-aa deletion in the TM4 domain of Pxα6 may exerts a global effect on nAChR conformation, rather than perhaps causing effects that are localised to the spinosad binding site.

The emergence of resistance to spinosad in insects does not typically confer cross-resistance to other classes of insecticides with different modes of action, however cross-resistance between spinosad and spinetoram might be expected (Sparks et al., 2012, Geng et al., 2013). In the present study, the spinosad-selected strain of *P. xylostella* developed 940-fold resistance to spinosad and also conferred an equivalent level of cross-resistance to spinetoram (1060-fold). In contrast, resistance did not extend to another macrocyclic lactone insecticide abamectin, which primarily acts at the glutamate-gated chloride channel (Wang et al., 2016b). Consequently, these findings are consistent with the evidence that abamectin has a different mode of action to that of spinosad or spinetoram (abamectin is classified by the Insecticide Resistance Action Committee as Mode of Action Group 6, whereas spinosad and spinetoram are Group 5). Although spinetoram is regarded as a next-generation spinosyn-based insecticide with improved efficacy, expanded spectrum and longer residual than spinosad, evidence of cross-resistance suggests that rotation of spinosad and spinetoram should be avoided in pest control programmes.

The most common type of spinosad resistance in insect pests (here referred to as type I resistance to spinosad) is characterized by high resistance (several hundred-fold), recessive inheritance, and target site modification (such as mutated nAChR α6 subunit gene). Type I resistance to spinosad has been reported for the Pear-Sel and SZ-SpinR strains of *P. xylostella* (Baxter et al., 2010, Rinkevich et al., 2010; this study), the spi-sel strain of *B. dorsalis* (Hsu et al., 2012), the R1S strain of *F. occidentalis* (Puinean et al., 2013), and the TS1 and TS5 strains of *T. palmi* (Bao et al., 2014). Spinosad resistance in laboratory-selected strains of *Heliothis virescens* and *Musca domestica* is of high level and recessive, and target site resistance mechanism is suggested but yet to be confirmed at molecular level (Roe et al., 2010, Wyss et al., 2003, Shono and Scott, 2003).

Although several studies have now associated target-site changes in the nAChR α6 subunit with resistance to spinosad, other mechanisms of resistance to spinosad have been reported. Studies of *M. domestica* suggest that resistance may be due to a recessive factor on autosome 1 (Shono and Scott, 2003) but appear to not be a consequence of target site changes in the nAChR α5, α6 or β3 subunits (Gao et al., 2007a, Gao et al., 2007b). Resistance to spinosad has been linked to enhanced detoxification in a variety of insect species, including the cotton bollworm *Helicoverpa armigera* (Wang et al., 2009) and *M. domestica* (Markussen and Kristensen, 2011). In addition, studies with the olive fly *Bactrocera oleae* have associated spinosad resistance to changes in energy metabolism genes (Sagri et al., 2014).

However, there may be more than one type of resistance to spinosad even for a particular pest species. Spinosad resistance in a field-derived strain of *P. xylostella* from Malaysia is inherited as a co-dominant trait (Sayyed et al., 2004), which differs from the type I resistance in both the Pear-Sel strain from Hawaii of the United States (Baxter et al., 2010) and the SZ-SpinR strain from Guangdong Province of China (this study). Although spinosad resistance is extremely high and recessive in both the R1S strain of *F. occidentalis* from Spain and the Spin-R strain from China, a point mutation of *Foα6* is the cause of spinosad resistance in R1S (Puinean et al., 2013), but *Foα6* has been reported to not be involved in resistance to spinosad in Spin-R (Hou et al., 2014). So, it will be critical to determine if the type I resistance is predominant in field populations of pests, and therefore adaptive resistance management strategies can be designed and implemented.

## Figures and Tables

**Table 1:** Toxicity of spinosad, spinetoram and abamectin to the susceptible (SZ) and resistant (SZ-SpinR) strains of *Plutella xylostella*.

Insecticide	Strain	N^a^	LC_50_ (95% FL^b^) (mg L^−1^)	Slope ± SD	RR^c^	*D*^d^
Spinosad	SZ (S)	240	0.27 (0.21–0.36)	1.71 ± 0.23		
	SZ-SpinR (R)	240	255 (182–369)	3.27 ± 0.51	940	
	F_1_ (R♂ × S♀)	240	0.84 (0.44–1.83)	3.35 ± 0.51	3.1	−0.67
	F_1_ (S♂ × R♀)	240	0.88 (0.63–1.28)	3.22 ± 0.49	3.3	−0.66
Spinetoram	SZ	240	0.037 (0.028–0.048)	2.05 ± 0.27		
	SZ-SpinR	240	39.2 (28.2–54.9)	3.41 ± 0.49	1060	
Abamectin	SZ	240	0.051 (0.040–0.065)	1.86 ± 0.20		
	SZ-SpinR	240	0.028 (0.017–0.067)	4.26 ± 0.58	0.5	

^a^ Number of larvae tested.

^b^ 95% Fiducial limits.

^c^ Resistance ratio = LC_50_ of SZ-SpinR or F_1_ progeny divided by LC_50_ of SZ.

^d^*D* values were calculated using the method of Stone (1968). *D* values can range from −1 (completely recessive) to 1 (completely dominant).

**Table 2:** Genetic linkage between a deletion mutation of Pxα6 and resistance to spinosad.

F_2_ progeny^a^	Number of individuals for each genotype^b^		
*rr*	*rS*	*SS*
Non-survivors (n = 47)	0	32	15
Survivors (n = 13)	13	0	0

^a^ F_1_ progeny between the susceptible SZ and resistant SZ-SpinR strains of *Plutella xylostella* were crossed to produce F_2_ progeny. Sixty larvae from the F_2_ progeny were treated with 10 mg L^−1^ of spinosad. Thirteen survivors and forty-seven non-survivors (dead larvae) were genotyped individually.

^b^*rr*: homozygous for the 9-bp deletion allele of *Pxα6*; *rS*: heterozygous for the 9-bp deletion allele of *Pxα6*; *SS*: homozygous for the wild type *Pxα6*.
